# Keep moving without hurting: The interaction between physical activity and pain in determining cognitive function at the population level

**DOI:** 10.1371/journal.pone.0197745

**Published:** 2018-06-01

**Authors:** Nelson Mauro Maldonato, Raffaele Sperandeo, Giovanni Caiazzo, Valeria Cioffi, Pasquale Cozzolino, Rosa Maria De Santo, Maria Luigia Fusco, Vittoria Silviana Iorio, Elena Gigante, Patrizia Marone, Nicole Nascivera, Chiara Scognamiglio

**Affiliations:** 1 Department of Neuroscience and Reproductive and Odontostomatological Sciences, University of Naples Federico II, Naples, Italy; 2 Department of Human Sciences, University of Basilicata, Potenza, Italy; 3 SiPGI, School of Specialization in Integrated Gestalt Psychotherapy, Torre Annunziata, Naples, Italy; Universidade Federal do Rio de Janeiro, BRAZIL

## Abstract

**Background:**

A number of studies have evaluated the association between cognitive function, pain, and physical activity. To our knowledge, however, no previous studies have evaluated these factors at the population level.

**Aims:**

To evaluate the association between cognitive function in the elderly with pain, physical activity, and the interaction between these variables. Estimates are generated for the United States population.

**Methods:**

We made use of the NHANES database (1999–2002), making adjustments so that our results represent the United States population. Cognitive function was evaluated through the Digit Symbol Substitution Test. Our main predictors were (1) pain, defined as soreness of either the shoulder, neck, lower back and joint, or a severe headache (2) physical activity, measured as the performance while performing tasks at home, physical activity intensity, walking, bicycle riding, and muscle strengthening.

**Results:**

Most individual pain sites were not significantly associated with cognitive function, while all physical activity factors were associated with an increase in cognitive function. When evaluating the sample subset of those with cognitive scores lower than the median, a combination of more pain and less physical activity was consistently associated with lower cognitive scores when compared to those performing more physical activity with or without pain. When evaluating individuals with cognitive scores above the median, a similar association pattern was perceived.

**Conclusions:**

Among the population of individuals above the age of 60, higher cognitive levels are associated with more physical activity and less with pain, although both factors might impact cognition. Public policy resources should be commensurate with these findings when targeting cognitive function among the aging population.

## Introduction

Identifying risk factors for decreased cognitive function in aging adults is of critical importance, as it not only directly impacts quality of life but also because cognitive loss can be an early sign of dementia. Despite knowing that physical inactivity and pain are independent risk factors for decreased cognitive function [1,2], to our knowledge there are no published populational estimates of their impact.

Decreased cognitive function has been associated with important negative health outcomes such as serious falls requiring hospitalization [3], Alzheimer’s disease, and death [4–6]. In addition, Alzheimer’s disease is also consistently preceded by a history of decreasing cognitive function [7,8]. Because a slight but detectable drop in cognitive function has been previously shown to predict dementia, cognition loss is considered a trait of preclinical dementia [9]. Although the role of early detection of cognitive deficits has been well established, the contribution of risk factors such as pain and physical activity on the development of cognitive dysfunctional at the populational level is less clear.

Evidence from animal [1,2], as well as human studies [10–14] have demonstrated that physical inactivity can increase the risk of early cognitive dysfunction. Specifically, exercise has a positive effect on the brain by regulating growth factors, which will ultimately act as mediators for brain stimulation [15]. In a similar manner, pain has been demonstrated to have a negative association with cognitive function [16,17]. Specifically, when aspects of cognitive function such as spatial discrimination, tactile acuity and learning curves are evaluated under painful situations, decreased cognition is consistently observed [18,19]. Furthermore, offering morphine over a long period of time to relieve chronic pain has led to improved cognitive function [20], thus confirming the role of pain in suppressing cognitive function [21,22]. Despite the fact that pain and physical inactivity having been shown to be independently associated with cognitive dysfunction, the co-existence of these two factors and their impact on cognition has not been previously evaluated at the population level.

In face of this gap in the literature, our objective was to determine whether co-existing, increasing levels of physical activity and decreased levels of pain were associated with increased cognitive function while making inferences to the United States population.

## Methods

### Study design

Our study was designed as a cross-sectional study based on NHANES (National Health and Nutrition Examination Survey) to evaluate the interaction between pain and physical activity in determining an increase in cognitive function at the population level. Cognitive function was evaluated through the Digit Symbol Substitution Test. Our analysis is described in accordance with the STROBE (STrengthening the Reporting of OBservational studies in Epidemiology) guidelines [23].

### Ethics

Our study was approved by the Institutional Review Board of the University of Basilicata, Italy.

### Setting

Data for this study were obtained from the National Health and Nutrition Examination Survey (NHANES) [24], a program that regularly assesses the health and nutritional status of citizens of the United States. This survey has been collecting data starting in the 1960s, with new data waves conducted every two years. For this study, we made use of the 1999–2000 and 2001–2002 year dyads, since both included a cognitive evaluation.

### Participants

Participants of the NHANES were drawn from 15 counties in the United States, selected through a stratified multistage probability sampling of the civilian non-institutionalized population. From a general group of over 5000 individuals interviewed and examined for various health conditions at their homes, we selected a subset involving all participants aged 60 and above. We also excluded all of those who could not complete the cognitive test, as well as individuals whose interviews were conducted by a proxy since the cognitive test would not represent the participant’s cognitive status.

### Outcomes

Our major outcome was cognitive function as measured by the total number of symbols a participant coded correctly within 120 seconds on the Digit Symbol Substitution Test (DSST), WAIS-III (Wechsler Adult Intelligence Scale, Third Edition) [25], and measured as the number of correctly identified items [26] (http://wwwn.cdc.gov/nchs/nhanes/1999-2000/CFQ.htm#References). The DSST is a non-verbal test of psychomotor speed and executive function [27–29], requiring response speed, visual-spatial skills, sustained attention, associative learning, and memory. This test is believed to be a more sensitive measure of dementia than the widely used Mini-Mental Status Exam [30], and is sensitive to cognitive changes at high levels of cognition [31].

### Predictors

Our main predictors were (1) pain, defined as the presence of shoulder pain, neck pain, lower back pain, joint pain, or severe headache; the evaluation of pain location as well as intensity were assessed by visual charts, and (2) physical activity, measured as the performance of home tasks, moderate or vigorous forms of physical activity, walking, riding a bicycle, and muscle-strengthening activities. All variables were obtained through a home interview including a cognitive exam.

### Potential confounders

Potential confounders were selected based on evidence from previous literature combined with clinical judgment. Specifically, we selected age, educational level, gender, household income, marital status, number people in a household, poverty index ratio, citizenship and the use of reading glasses. [32].

### Statistical methods

Our exploratory analysis started by evaluating distributions, frequencies, and percentages for each of the numeric and categorical variables. Categorical variables were evaluated for near-zero variation [33], or categories with low frequencies which could bias our results. Extensive graphical displays were used for both univariate analysis and bivariate associations. Missing data were explored using a combination of graphical displays involving univariate, bivariate and multivariate methods. For bivariate analyses, we calculated effect sizes to quantify the association between DSST scores and sample characteristics such as age, gender, income, education level, marital status, and citizenship. Specifically, for t-tests we used Cohen’s d statistic interpreted as being small when d < = 0.2, medium if d < = 0.5, and 0.8 as a ‘large’ effect size [34]. We used Cohen’s w, a measure of effect size for Chi-square tests, with values of 0.1, 0.3, and 0.5 representing small, medium, and large effect sizes, respectively [34].

Our modeling strategy made use of a series of generalized linear models with a Gaussian family to evaluate the association between cognitive function measured by the processing speed evaluated through the DSST, pain (presence of shoulder pain, neck pain, lower back pain, joint pain or severe headache), and physical activity (measured as the performance of home tasks, moderate or vigorous forms of physical activity, walking, riding a bicycle, and muscle-strengthening activities). Each of these variables were added as indicator variables in our model. The multiplicative interaction between these variables was evaluated through the generation of indicator variables (dummy variables) representing the presence of either any pain or any indicator of physical activity. Risk-adjusted models took into account the following potential confounding variables: age, educational level, gender, household income, marital status, number people in a household, poverty index ratio, citizenship and the use of reading glasses. Results were reported as predicted means with 95% confidence intervals, with results being interpreted as significant when confidence intervals did not overlap.

All of our analyses were adjusted by using weights and strata as specified in the NHANES sampling strategy, so that our results would ultimately represent inferences to the United States population rather than just the local study sample. All analyses were performed using the R language [35] and the following packages: svy (https://cran.r-project.org/web/packages/survey/index.html), ggplot2 (https://cran.r-project.org/web/packages/ggplot2/index.html), and rmarkdown (https://cran.r-project.org/web/packages/rmarkdown/index.html).

## Results

Following the merging of data from the 1999–2000 and 2001–2002 surveys, 2,975 participants were included in our analysis. Table 1 displays the description of the overall study sample stratified by the median value of the normally distributed Digit Symbol Substitution Test (DSST) Score, measuring processing speed as a component of cognitive ability. Numeric variables were compared through t-tests and categorical variables were compared though Chi-square tests. The average age of participants was 71.63 (± 7.96) and the female-male ratio was approximately 1:1. Participants who were female (55.1%, Cohen’s w = 0.068), younger (69 yrs vs. 73 yrs, Cohen’s d = 0.1), married (63.5%, Cohen’s w = 0.16), and US citizens (98%, Cohen’s w = 0.16) presented significantly higher scores, indicating higher cognitive levels (p < 0.001) when compared with participants with lower cognitive scores. Based on Cohen’s classification for effect sizes, this difference was classified as small despite its statistically significance. High household income (> 45,000–40.4%, Cohen’s w = 0.32) and high school education level (24.9%, Cohen’s w = 0.47) presented a significant association with upper median DSST scores (p < 0.001), indicating medium and large effect sizes, respectively, when comparing the groups with above-median DSST scores versus those with lower than median scores. An increased Metabolic Equivalent of Task (MET) score, a measure of the energy expenditure related to physical activities, was significantly associated with upper median cognitive scores, demonstrating a significantly increased DSST scores when compared with those with DSST scores lower than the median (p < 0.001). This association also presented a small effect size (d = 0.16).

**Table 1:** Characteristics of study population stratified by Upper Quartile Digit Symbol Substitution Test Score.

Variable [Missing]	Total (2975)	Lower median DSST scores (1505)	Upper median DSST scores (1470)	p [effect size]
Age [0]	71.6 (± 7.96)	73.5 (± 8.08)	69.7 (± 7.37)	p < 0.001 [0.1]
Education Level [7]				p < 0.001 [0.47]
- Graduate	466 (15.7%)	100 (6.67%)	366 (24.9%)	
- High School	728 (24.5%)	299 (19.9%)	429 (29.2%)	
- Incomplete High School	514 (17.3%)	331 (22.1%)	183 (12.5%)	
- Middle School	676 (22.7%)	587 (39.2%)	89 (6.06%)	
- Undergraduate	584 (19.6%)	182 (12.1%)	402 (27.4%)	
Female [0]	1,537 (51.7%)	727 (48.3%)	810 (55.1%)	p < 0.001 [0.068]
Household Income Categories [418]				p < 0.001 [0.32]
- < = 20,000	922 (31%)	635 (49.2%)	287 (22.7%)	
- < = 45,000	918 (30.9%)	450 (34.9%)	468 (36.9%)	
- > 45,000	717 (24.1%)	205 (15.9%)	512 (40.4%)	
Marital Status [145]				p < 0.001 [0.16]
- Divorced	245 (8.24%)	120 (8.45%)	125 (8.87%)	
- Living With Partner	35 (1.18%)	13 (0.92%)	22 (1.56%)	
- Married	1,681 (56.5%)	748 (52.7%)	933 (66.2%)	
- Never Married	78 (2.62%)	49 (3.45%)	29 (2.06%)	
- Separated	59 (1.98%)	42 (2.96%)	17 (1.21%)	
- Widowed	732 (24.6%)	448 (31.5%)	284 (20.1%)	
Metabolic Equivalent of Task [METs] Score [1,650]	8.35 (± 6.71)	7 (± 5.2)	9.18 (± 7.37)	p < 0.001 [0.16]
Number People in the Household [0]	2.26 (± 1.32)	2.37 (± 1.49)	2.14 (± 1.11)	p < 0.001 [0.1]
Poverty Index Ratio [377]	2.53 (± 1.54)	1.93 (± 1.29)	3.16 (± 1.52)	p < 0.001 [0.11]
US Citizen [6]	2,794 (93.9%)	1,354 (90%)	1,440 (98%)	p < 0.001 [0.16]
Wear Glasses To Read [0]	2,141 (72%)	1,121 (74.5%)	1,020 (69.4%)	p = 0.002 [0.0567]

Table 2 demonstrates the association between predictors and DSST scores measuring cognition function, evaluated through a multiple linear regression model and displayed as predicted means with 95% confidence intervals. Results were considered statistically significant when confidence intervals did not overlap between different estimates. When evaluating the impact of individual pain sites on cognitive function, only left shoulder pain was significantly associated with low DSST scores, indicating decreased cognitive levels [Predicted mean 42.74, 95% CI (40.05, 45.44) vs. 46.76, 95% CI (45.48, 48.04), R-squared = 0.46].

**Table 2:** Unadjusted association between cognitive function and individual pain site.

Variables	DSST* scores
Shoulder Pain	
- Absent	46.76 (45.46, 48.06)
- Present	43.75 (41.33, 46.16)
Left Shoulder Pain	
- Absent	46.76 (45.48, 48.04)
- Present	42.74 (40.05, 45.44)
Right Shoulder Pain	
- Absent	46.73 (45.45, 48.02)
- Present	43.12 (40.2, 46.03)
Neck Pain	
- Absent	46.89 (45.49, 48.28)
- Present	43.5 (41.23, 45.77)
Low Back Pain	
- Absent	46.8 (45.22, 48.39)
- Present	45.41 (43.77, 47.05)
Severe Headaches	
- Absent	46.56 (45.22, 47.91)
- Present	43.82 (40.93, 46.71)
Joint Pain	
- Absent	47.09 (45.58, 48.6)
- Present	45.52 (43.77, 47.28)

* DSST—Digit Symbol Substitution Test

In contrast, while evaluating the association between physical activity and cognitive function through DSST scores, we observed that all isolated forms of physical activity were significantly associated with high DSST scores, indicating increased cognitive function since all confidence intervals for predicted means were non-overlapping (R square = 0.42) (Table 3).

**Table 3:** Unadjusted association between cognition and physical activity.

Variables	DSST* scores
Performs Home Tasks	
- No	40.76 (39.32, 42.2)
- Yes	51.15 (49.63, 52.68)
Performs Vigorous Activities	
- No	44.61 (43.4, 45.82)
- Yes	54.04 (51.77, 56.31)
Performs Moderate Activities	
- No	42.34 (40.96, 43.71)
- Yes	51.59 (50.07, 53.12)
Performs Walk or Rides a Bicycle	
- No	45.95 (44.59, 47.3)
- Yes	47.43 (45.18, 49.69)
Performs Muscle Strengthening Activities	
- No	45.02 (43.81, 46.23)
- Yes	53.02 (51.2, 54.83)

*DSST—Digit Symbol Substitution Test

When evaluating the adjusted interaction between pain and physical activity in determining cognitive function, we stratified our results by individuals in the lower 33rd percentile and those in the upper 77th percentile of the DSST. Lower DSST scores indicate decreased cognitive function and higher scores are associated with increased cognitive function. More pain and less physical activity were consistently associated with lower cognitive scores than those involved in more physical activities and/or having less pain (R-square = 0.56). There was no significant difference, however, in relation to those who presented less pain. When evaluating those in the upper cognitive score stratum, a similar association was observed. (Table 4).

**Table 4:** Cognitive function and adjusted interaction between pain and physical activity.

Pain and physical activity	Lower DSST* score	Upper DSST score
High pain and low physical activity levels	15.91 (13.46, 18.36)	44.4 (42.56, 46.24)
Low pain and low physical activity levels	15.46 (12.55, 18.37)	45.2 (42.64, 47.76)
Low pain and high physical activity levels	17.41 (14.75, 20.08)	48.49 (46.58, 50.4)
High pain and high physical activity levels	17.49 (14.72, 20.25)	46.72 (44.66, 48.78)

*DSST—Digit Symbol Substitution Test

**Fig 1:**
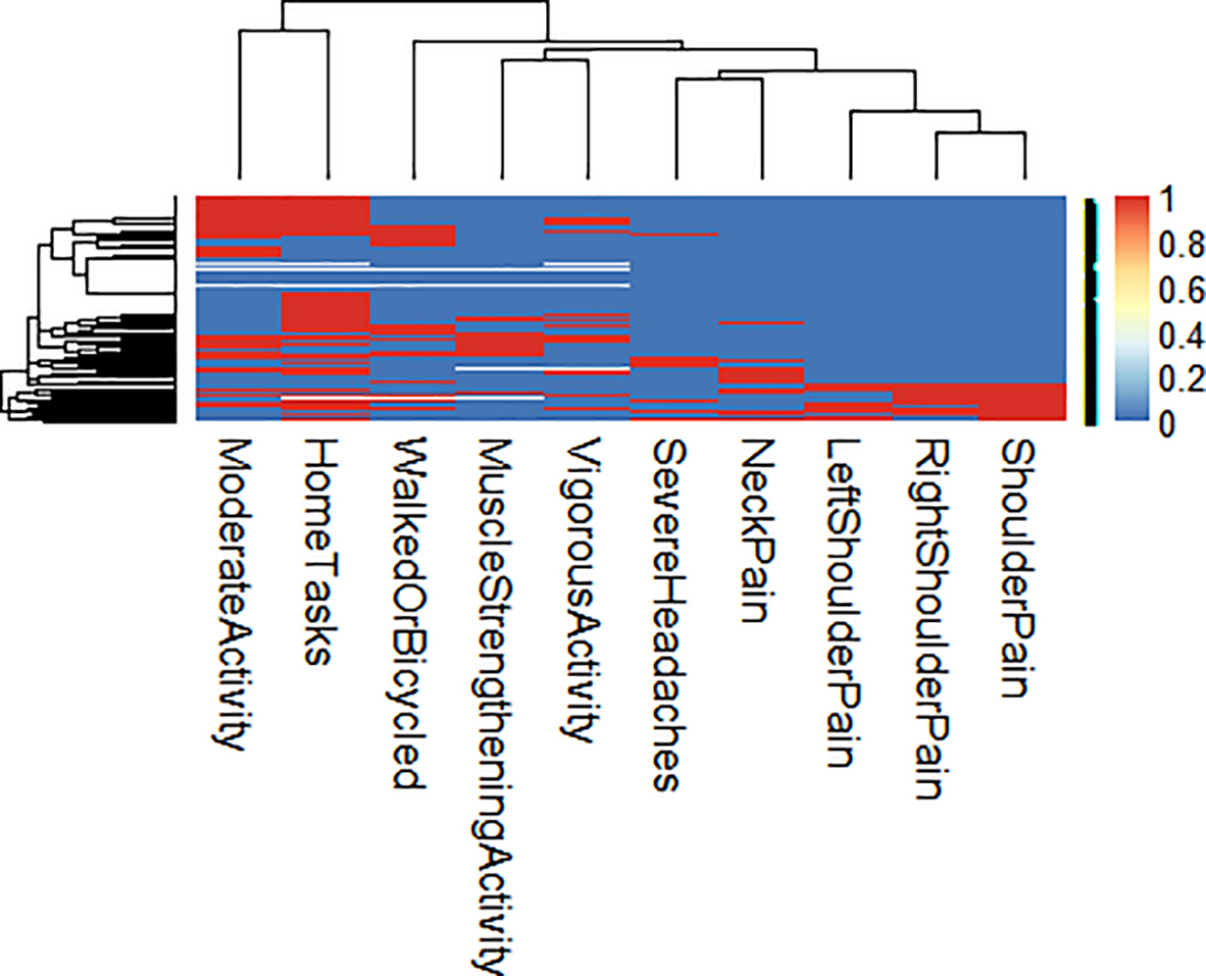
Higher cognitive function.

**Fig 2:**
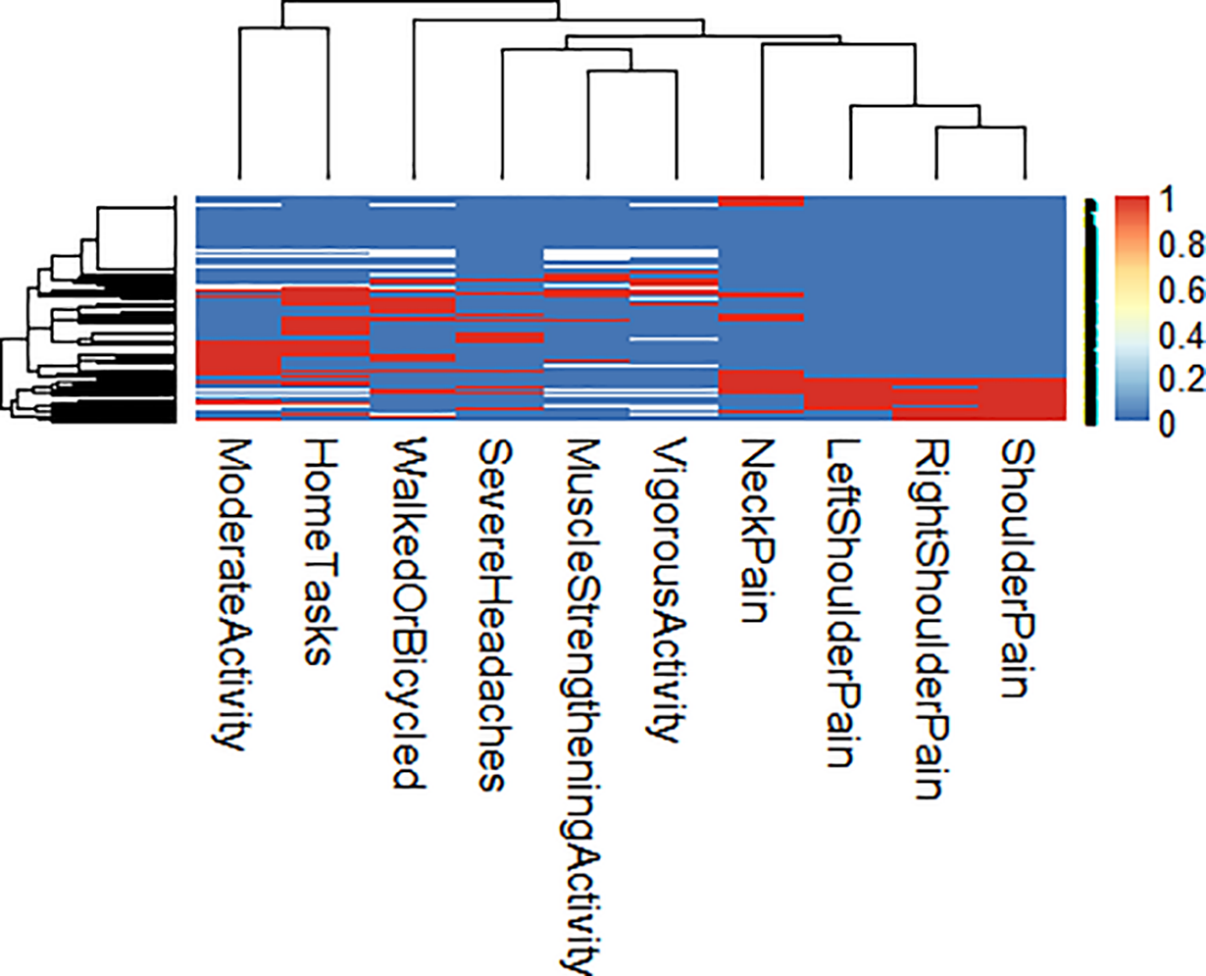
Lower cognitive function.

Finally, we made use of heatmaps to compare the impact of individual pain sites and physical activity on cognitive function among those in the lower 33th percentile of the DSST scores. The heatmap demonstrates how different predictors cluster in relation to DSST scores. This analysis validated our previous results showing that, in general, higher levels of cognitive function were associated with the whole spectrum of physical activity as demonstrated by the more homogeneous distribution of red stripes, while pain presented a smaller role as showed by the concentration of red stripes at the bottom of the heatmap (Fig 1and Fig 2).

## Discussion

To our knowledge, this is the first study evaluating the interaction between pain and physical activity in determining cognitive function evaluated through DSST scores and while also making inferences at the population level. We found that most individual pain sites were not significantly associated with cognitive function in the overall population, while all physical activity factors were associated with an increase in cognitive function. When splitting the population among those within the lower 33th percentile of cognitive scores, more pain and less physical activity were consistently associated with lower cognitive scores when compared with those performing more physical activity regardless of their pain level as well as compared to those performing more physical activity and having less pain. There was no significant difference, however, in relation to those who just presented less pain. When evaluating those with the upper cognitive scores, a similar association pattern was observed. In summary, physical activity were invariably beneficial, while low pain levels were only beneficial to those with already low cognitive levels. In addition, while the effects of decreased levels of pain and increased levels of physical activity were synergistic, there was “no additional bonus,” demonstrated by a multiplicative interaction of simultaneously having low pain levels and high physical activity levels.

The International Association for the Study of Pain (IASP) describes pain as “the unpleasant sensory or emotional experience associated with actual or potential tissue damage, or described in terms of such damage” [36]. Pain is therefore essentially a construct affecting both physiological and psychological states of an individual. While up to a quarter of the population experiences moderate to severe pain, most do not receive adequate treatment [37]. Because pain and cognition have overlapping pathways with pain perception partially depending on cognitive evaluation through learning, memory, and decision making [38,39], an association between them would not be surprising. This association has been extensively studied. Both pre-clinical [40,41] and clinical studies [21,42] considering pain and various aspects of cognitive function are in support of the concept of increased pain levels being associated with reduced cognitive function. Some studies, however, failed to confirm this relationship, which partially aligns with our results [43,44]. Of importance, we believe that while our study does not refute the concept of pain being a component affecting cognitive levels, it supports the idea that at the population level pain does not have the same level of importance as physical activity. This finding can likely be explained since most of the general population over the age of 60 does not undergo pain levels large enough to affect cognitive functioning.

Similarly, there is strong evidence supporting the association between physical inactivity and loss of cognitive function [1,15,45–49], physical inactivity having been previously associated with reduced cognitive levels. Several mechanisms may explain this relationship. Physical activity might improve the brain’s vascular condition by lowering blood pressure, adjusting lipoprotein profile, increasing cerebral endothelial nitric oxide production [50], and increasing cerebral blood flow [51]. In addition, physical activity may improve cognition by enhancing the expansion of brain cells, forming new synapses, and inducing neovascularization [52]. Some studies, however, only found a weak association between cognitive function and physical activity, likely due to the intensity of physical activity in these studies not covering a wide enough range [53].

Despite filling an important gap in the literature, our study does have limitations. First, we only made use of a single cognitive test, which limits the scope of our cognitive evaluation. Despite its simplicity, the Digit Symbol Substitution Test from the Wechsler Adult Intelligence Scale-Third Edition (WAIS-III) covers a number of associated domains considered important for the day-to-day function and well-being in the general population. Second, our latest data covers the late 1990s and early 2000s, and since that time overall populational levels of both pain and physical activity might have changed. We would argue, however, that although this modification might have been quantitative, it is less likely that the overall nature of the association might have changed. In other words, although the levels of cognitive impairment might have increased as a function of the progressive aging of the American population, the association might still hold true for specific pain, physical activity, and cognitive function levels.

In conclusion, our study emphasizes the importance of ongoing, population-level campaigns and healthcare policies providing incentives for an increase in physical activity levels among the general population as well as the elderly. Despite a weaker association, our findings also emphasize the importance of controlling pain levels at the population level.

## References

[1] Black JE, Isaacs KR, Anderson BJ, Alcantara AA, Greenough WT. Learning causes synaptogenesis, whereas motor activity causes angiogenesis, in cerebellar cortex of adult rats. Proc Natl Acad Sci. 1990;87: 5568–5572. doi: 10.1073/pnas.87.14.5568 1695380 10.1073/pnas.87.14.5568 PMC54366

[2] Praag H van, Christie BR, Sejnowski TJ, Gage FH. Running enhances neurogenesis, learning, and long-term potentiation in mice. Proc Natl Acad Sc. 1999;96: 13427–13431. doi: 10.1073/pnas.96.23.13427 10557337 10.1073/pnas.96.23.13427 PMC23964

[3] Welmerink DB, Longstreth W, Lyles MF, Fitzpatrick AL. Cognition and the risk of hospitalization for serious falls in the elderly: Results from the cardiovascular health study. The J Gerontol A Biol Sci Med Sci. 2010;65: 1242–1249. doi: 10.1093/gerona/glq115 20584769 10.1093/gerona/glq115 PMC2954237

[4] Bennett DA, Wilson RS, Schneider JA, Evans DA, Beckett LA, Aggarwal NT, et al Natural history of mild cognitive impairment in older persons. Neurology. 2002;59: 198–205. doi: 10.1212/WNL.59.2.198 12136057 10.1212/wnl.59.2.198

[5] Swindell WR, Cummings SR, Sanders JL, Caserotti P, Rosano C, Satterfield S, et al Data mining identifies digit symbol substitution test score and serum cystatin C as dominant predictors of mortality in older men and women. Rejuvenation Res. 2012;15: 405–413. doi: 10.1089/rej.2011.1297 22607624 10.1089/rej.2011.1297 PMC3419847

[6] Newman AB, Sachs MC, Arnold AM, Fried LP, Kronmal R, Cushman M, et al Total and cause-specific mortality in the cardiovascular health study. Gerontol A Biol Sci Med Sci. 2009;64: 1251–1261. 10.1093/gerona/glp127 PMC2773812 19723772

[7] Small BJ, Fratiglioni L, Viitanen M, Winblad B, Bäckman L. The course of cognitive impairment in preclinical Alzheimer disease: Three- and 6-year follow-up of a population-based sample. Arch Neurol. 2000;57: 839–844. doi: 10.1001/archneur.57.6.839 10867781 10.1001/archneur.57.6.839

[8] Kawas CH, Corrada MM, Brookmeyer R, Morrison A, Resnick SM, Zonderman AB, et al Visual memory predicts Alzheimer’s disease more than a decade before diagnosis. Neurology. 2003;60: 1089–1093. doi: 10.1212/01.WNL.0000055813.36504.BF 12682311 10.1212/01.wnl.0000055813.36504.bf

[9] Linn RT, Wolf PA, Bachman DL, et al The ‘preclinical phase’ of probable Alzheimer’s disease: A 13-year prospective study of the Framingham cohort. Arch Neurol. 1995;52: 485–490. doi: 10.1001/archneur.1995.00540290075020 7733843 10.1001/archneur.1995.00540290075020

[10] Weuve J, Kang J, Manson JE, Breteler MB, Ware JH, Grodstein F. Physical activity, including walking, and cognitive function in older women. JAMA. 2004;292: 1454–1461. doi: 10.1001/jama.292.12.1454 15383516 10.1001/jama.292.12.1454

[11] Berkman LF, Seeman TE, Albert M, Blazer D, Kahn R, Mohs R, et al High, usual and impaired functioning in community-dwelling older men and women: Findings from the MacArthur Foundation Research Network on successful aging. J Clin Epidemiol. 1993;46: 1129–1140. doi: 10.1016/0895-4356(93)90112-E 8410098 10.1016/0895-4356(93)90112-e

[12] Hultsch DF, Hammer M, Small BJ. Age differences in cognitive performance in later life: relationships to self-reported health and activity life style. J Gerontol. 1993;48: P1–P11. doi: 10.1093/geronj/48.1.P1 8418144 10.1093/geronj/48.1.p1

[13] Colcombe S, Kramer AF. Fitness effects on the cognitive function of older adults: a meta-analytic study. Psychol Sci. 2003;14: 125–130. doi: 10.1111/1467-9280.t01-1-01430 12661673 10.1111/1467-9280.t01-1-01430

[14] Clarkson-Smith L, Hartley AA. Structural equation models of relationships between exercise and cognitive abilities. Psychol Aging. 1990;5: 437–446. doi: 10.1037/0882-7974.5.3.437 2242248 10.1037//0882-7974.5.3.437

[15] Gómez-Pinilla F, Dao L, So V. Physical exercise induces FGF-2 and its mRNA in the hippocampus. Brain Res. 1997;764: 1–8. doi: 10.1016/S0006-8993(97)00375-2 9295187 10.1016/s0006-8993(97)00375-2

[16] Lee DM, Pendleton N, Tajar A, O’Neill TW, O’Connor DB, Bartfai G, et al Chronic widespread pain is associated with slower cognitive processing speed in middle-aged and older European men. Pain. 2010;151: 30–36. doi: 10.1016/j.pain.2010.04.024 20646831 10.1016/j.pain.2010.04.024

[17] Grace GM, Nielson WR, Hopkins M, Berg MA. Concentration and memory deficits in patients with fibromyalgia syndrome. J Clin Exp Neuropsychol. 1999;21: 477–487. doi: 10.1076/jcen.21.4.477.876 10550807 10.1076/jcen.21.4.477.876

[18] Maihöfner C, DeCol R. Decreased perceptual learning ability in complex regional pain syndrome. Eur J Pain. 2007;11: 903–909. doi: 10.1016/j.ejpain.2007.03.006 17451979 10.1016/j.ejpain.2007.03.006

[19] Verdejo-García A, López-Torrecillas F, Calandre EP, Delgado-Rodríguez A, Bechara A. Executive function and decision-making in women with fibromyalgia. Arch Clin Neuropsychol. 2009;24: 113–122. doi: 10.1093/arclin/acp014 19395361 10.1093/arclin/acp014

[20] Tassain V, Attal N, Fletcher D, Brasseur L, Dégieux P, Chauvin M, et al Long term effects of oral sustained release morphine on neuropsychological performance in patients with chronic non-cancer pain. Pain. 2003;104: 389–400. doi: 10.1016/S0304-3959(03)00047-2 12855350 10.1016/s0304-3959(03)00047-2

[21] Oosterman JM, Derksen LC, Wijck AJ van, Veldhuijzen DS, Kessels RP. Memory functions in chronic pain: examining contributions of attention and age to test performance. Clin J Pain. 2011;27: 70–75. doi: 10.1097/AJP.0b013e3181f15cf5 20842018 10.1097/AJP.0b013e3181f15cf5

[22] Moriarty O, McGuire BE, Finn DP. The effect of pain on cognitive function: A review of clinical and preclinical research. Prog Neurobiol. 2011;93: 385–404. doi: 10.1016/j.pneurobio.2011.01.002 21216272 10.1016/j.pneurobio.2011.01.002

[23] Elm E von, Altman DG, Egger M, Pocock SJ, Gøtzsche PC, Vandenbroucke JP, et al Strengthening the Reporting of Observational Studies in Epidemiology (STROBE) statement: guidelines for reporting observational studies. PLOS Med. 2007;4: e296 doi: 10.1371/journal.pmed.0040296 17941714 10.1371/journal.pmed.0040296 PMC2020495

[24] Centers for Disease Control and Prevention (CDC). National Center for Health Statistics (NCHS). National Health and Nutrition Examination Survey Data. Hyattsville, MD: U.S. Department of Health and Human Services, Centers for Disease Control and Prevention Available: https://wwwn.cdc.gov/nchs/nhanes/Default.aspx

[25] Kaufman AS, Lichtenberger EO. Essentials of WAIS-III assessment. John Wiley & Sons Inc; 1999.

[26] NHANES 1999–2000: Cognitive Functioning Data documentation, codebook, and frequencies. Available: http://wwwn.cdc.gov/nchs/nhanes/1999-2000/CFQ.htm#References

[27] Sperandeo R, Maldonato NM, Baldo G, Dell’Orco S. Executive functions, temperament and character traits: A quantitative analysis of the relationship between personality and prefrontal functions. Cognitive infocommunications (coginfocom), 2016 7th IEEE International Conference on. IEEE; 2016. pp. 000043–000048.

[28] Sperandeo R, Moretto E, Baldo G, Dell’Orco S, Maldonato NM. Executive functions and personality features: A circular interpretative paradigm. Cognitive infocommunications (coginfocom), 2017 8th IEEE International Conference on. IEEE; 2017. pp. 000063–000066.

[29] Maldonato NM, Oliverio A, Esposito A. Neuronal symphonies: Musical improvisation and the centrencephalic space of functional integration. World Futures. 2017; 1–20.

[30] Lin FR, Yaffe K, Xia J, Xue Q-L, Harris TB, Purchase-Helzner E, et al Hearing loss and cognitive decline in older adults. JAMA Intern Med. 2013;173 doi: 10.1001/jamainternmed.2013.1868 23337978 10.1001/jamainternmed.2013.1868 PMC3869227

[31] Proust-Lima C, Amieva H, Dartigues J-F, Jacqmin-Gadda H. Sensitivity of four psychometric tests to measure cognitive changes in brain aging-population–based studies. Am J Epidemiol. 2006;165: 344–350. doi: 10.1093/aje/kwk017 17105962 10.1093/aje/kwk017 PMC2244646

[32] Lee PH. Should we adjust for a confounder if empirical and theoretical criteria yield contradictory results? A simulation study. Sci Rep. 2014;4 doi: 10.1038/srep06085 25124526 10.1038/srep06085 PMC5381407

[33] Kuhn M, Johnson K. Applied predictive modeling 2013 Springer. ISBN-13.

[34] Ellis PD. The essential guide to effect sizes: Statistical power, meta-analysis, and the interpretation of research results Cambridge University Press; 2010.

[35] R Core Team. R: A language and environment for statistical computing Vienna, Austria: R Foundation for Statistical Computing; 2015 Available: http://www.R-project.org/

[36] Mersky H, Bodguk N. Task force on taxonomy of the international association for the study of pain: Classification of chronic pain, description of chronic pain syndromes and definition of pain terms Seattle, WA: IASP Press; 1994.

[37] Breivik H, Collett B, Ventafridda V, Cohen R, Gallacher D. Survey of chronic pain in Europe: Prevalence, impact on daily life, and treatment. Eur J Pain. 2006;10: 287–287. doi: 10.1016/j.ejpain.2005.06.009 16095934 10.1016/j.ejpain.2005.06.009

[38] Maldonato NM, Dell’Orco S. How to make decisions in an uncertain world: Heuristics, biases, and risk perception. World Futures. 2011;67: 569–577.

[39] Maldonato NM, Dell’Orco S, Sperandeo R. When intuitive decisions making, based on expertise, may deliver better results than a rational, deliberate approach. Multidisciplinary approaches to neural computing. 2018. pp. 369–377.

[40] Boyette-Davis J, Thompson C, Fuchs P. Alterations in attentional mechanisms in response to acute inflammatory pain and morphine administration. Neuroscience. 2008;151: 558–563. doi: 10.1016/j.neuroscience.2007.10.032 18065152 10.1016/j.neuroscience.2007.10.032

[41] Millecamps M, Etienne M, Jourdan D, Eschalier A, Ardid D. Decrease in non-selective, non-sustained attention induced by a chronic visceral inflammatory state as a new pain evaluation in rats. Pain. 2004;109: 214–224. doi: 10.1016/j.pain.2003.12.028 15157681 10.1016/j.pain.2003.12.028

[42] Lee D, Pendleton N, Tajar A, O’Neill T, O’Connor D, Bartfai G, et al Chronic widespread pain is associated with slower cognitive processing speed in middle-aged and older European men. Pain. 2010;151: 30–36. doi: 10.1016/j.pain.2010.04.024 20646831 10.1016/j.pain.2010.04.024

[43] Karp JF, Reynolds CF, Butters MA, Dew MA, Mazumdar S, Begley AE, et al The relationship between pain and mental flexibility in older adult pain clinic patients. Pain Med. 2006;7: 444–452. doi: 10.1111/j.1526-4637.2006.00212.x 17014605 10.1111/j.1526-4637.2006.00212.x PMC2946642

[44] Antepohl W, Kiviloog L, Andersson J, Gerdle B. Cognitive impairment in patients with chronic whiplash-associated disorder-a matched control study. NeuroRehabilitation. 2003;18: 307–316. 14757927

[45] Neeper SA, Gómez-Pinilla F, Choi J, Cotman CW. Brain Res. 1996;726: 49–56. 8836544

[46] Clarkson-Smith L, Hartley AA. Structural equation models of relationships between exercise and cognitive abilities. Psychol Aging. 1990;5: 437 2242248 10.1037//0882-7974.5.3.437

[47] Albert MS, Jones K, Savage CR, Berkman L, Seeman T, Blazer D, et al Predictors of cognitive change in older persons: MacArthur studies of successful aging. Psychol Aging. 1995;10: 578 8749585 10.1037//0882-7974.10.4.578

[48] Laurin D, Verreault R, Lindsay J, MacPherson K, Rockwood K. Physical activity and risk of cognitive impairment and dementia in elderly persons. Arch Neurol. 2001;58: 498–504. 11255456 10.1001/archneur.58.3.498

[49] Williams P, Lord SR. Effects of group exercise on cognitive functioning and mood in older women. Aust N Z J Public Health. 1997;21: 45–52. 9141729 10.1111/j.1467-842x.1997.tb01653.x

[50] Taddei S, Galetta F, Virdis A, Ghiadoni L, Salvetti G, Franzoni F, et al Physical activity prevents age-related impairment in nitric oxide availability in elderly athletes. Circulation. Am Heart Assoc; 2000;101: 2896–2901. 10869260 10.1161/01.cir.101.25.2896

[51] Rogers RL, Meyer JS, Mortel KF. After reaching retirement age physical activity sustains cerebral perfusion and cognition. J Am Geriatr Soc. 1990;38: 123–128. 2299115 10.1111/j.1532-5415.1990.tb03472.x

[52] Chodzko-Zajko WJ, Moore KA. Physical fitness and cognitive functioning in aging. Exerc Sport Sci Rev. 1994;22: 195–220. 7925543

[53] Blumenthal JA, Emery CF, Madden DJ, Schniebolk S, Walsh-Riddle M, George LK, et al Long-term effects of exercise on psychological functioning in older men and women. J Gerontol. 1991;46: P352–P361. 1940092 10.1093/geronj/46.6.p352

